# A Duplication Upstream of *SOX9* Associated with *SRY* Negative 46,XX Ovotesticular Disorder of Sex Development: A Case Report

**DOI:** 10.4274/jcrpe.galenos.2019.2019.0101

**Published:** 2020-09-02

**Authors:** Eda Mengen, Gülsüm Kayhan, Pınar Kocaay, Seyit Ahmet Uçaktürk

**Affiliations:** 1 Ankara City Hospital, Children’s Hospital, Clinic of Pediatric Endocrinology, Ankara, Turkey; 2 Gazi University Faculty of Medicine, Department of Medical Genetics, Ankara, Turkey

**Keywords:** 46,XX ovotesticular disorder of sex development, SRY-negative, SOX9

## Abstract

The 46,XX ovotesticular disorder of sex development (DSD) is rarely observed in humans. This disorder is generally described as ambiguous genitalia with the presence of ovarian and testicular tissues in different gonads or in the same gonad. Almost no subjects with 46,XX ovotesticular DSD have sex-determining region of the Y chromosome *(SRY)* gene. It is known that excessive expression of *SRY*-related high mobility group box 9 *(SOX9)* is the cause of *SRY*-negative 46,XX ovotesticular DSD in the absence of *SRY*. Here, we analyzed our *SRY*-negative case with 46,XX ovotesticular DSD. In an array comparative genomic hybridization study using a peripheral blood sample from the patient, a duplication of 1114 kb (Hg19 coordinates: chr17:69006280-70120619) in the region of 17q24.3 containing *SOX9* was detected. This is the first case reported from Turkey, exhibiting *SOX9* duplication in *SRY*-negative 46,XX ovotesticular DSD.

**What is already known on this topic?**

The 46,XX ovotesticular disorder of sex development (DSD) is rarely observed in humans. It is known that excessive expression of *SRY*-related high mobility group box 9 *(SOX9)* is the cause of SRY-negative 46,XX ovotesticular DSD in the absence of *SRY*.

**What this study adds?**

This is the first case reported in Turkey, exhibiting SOX9 duplication in a patient with SRY-negative 46,XX ovotesticular DSD.

## Introduction

Normal gonadal differentiation and sex development depend on the synchronization of the pathways reflecting the effects and interactions of certain genes, transcription factors, and hormones in the genetic sex presence determined by the chromosomal structure. First, ovarian or testicular development from a primitive gonad occurs and then differentiation of internal and external genital structures take place. Modifications of these complex gene regulatory networks or deterioration of the gene expression regulating fetal gonadal development lead to disorders of sex development (DSD) (1,2).

These disorders include a heterogeneous abnormality spectrum in which the chromosomal, genetic, gonadal, hormonal or phenotypical aspects of sex are atypical. The gap in terminology provides a frame for approaching differential diagnosis in a patient. DSD categories include sex chromosome DSDs, such as 45,X/46,XY; ovotesticular DSD; 46,XY DSDs, such as disorders of testicular development, disorders of androgen synthesis and action, and XY sex reversal; and 46,XX DSDs such as masculinization of the XX individual and XX sex reversal. The incidence of DSDs is approximately 1 in every 4000 infants (1).

Usually ovarian differentiation proceeds in the normal way in the 46,XX fetus (1). However, in rare cases, 46,XX gonads can either differentiate in testes, which is known as 46,XX testicular DSD (previously termed XX male), or may cause a condition causing ovarian and testicular tissue to couple in the same individual, known as 46,XX ovotesticular DSD (previously termed true hermaphroditism) (3). Ovotesticular DSD is a rare DSD in which an individual generally has ambiguous genitalia and ovarian and testicular tissues are present in separate gonads or in the same gonad; two third of these have the 46,XX karyotype. Molecular studies have revealed that almost 90% of 46,XX patients with ovotesticular DSD were negative for the sex-determining region of the Y chromosome (*SRY*) gene (4). In these *SRY* negative individuals, testicular development may depend on the presence of an additional dose or excessive expression of an autosomal gene which influences male sexual differentiation such as SRY-related high mobility group box 9 (*SOX9*) (5).

To date, the definitive mechanism of testicular differentiation in 46,XX ovotesticular DSD has not been explained. Three putative mechanisms have been proposed to explain testicular determination: (i) Hidden mosaicism with a cell line carrying Y; (ii) translocation of Y-material from paternal Y to the X chromosome including the *SRY* gene; (iii) in a gene which is autosomal or connected to X, defects allowing testicular determination in the absence of *SRY* - *SOX9* is one of these genes (4).

*SOX9* duplication is not a common cause of testicular development in cases with *SRY*-negative 46,XX testicular or ovotesticular DSD (6). Only three studies have been published exhibiting *SOX9* duplication in *SRY*-negative 46,XX ovotesticular DSD (4,5,7). This study is the first case reported from Turkey, exhibiting *SOX9* duplication in *SRY*-negative 46,XX ovotesticular DSD. In addition, a literature review of *SRY*-negative 46,XX ovotesticular DSD is presented and the role of *SRY* and *SOX9* in testicular development is discussed.

## Case Report

A three-year and four-month-old male patient was brought due to uncertainty in the genital region. The patient was born through normal vaginal delivery with a birth weight of 3450 g, equivalent to a standard deviation score (SDS) of 0.28, and a birth length of 50 cm (-0.05 SDS) in the 39^th^ gestational week, and this was the 4^th^ pregnancy of the 32-year-old, healthy mother. The patient, whose atypical genitalia was discovered after birth, was examined because of possible DSD in an external center. The parents are non‑consanguineous and family members exhibited no clinical manifestations. The family is of Turkish origin. Peripheral blood chromosome analysis was assessed as 46,XX. Medical history when he was 36 days old, showed: serum testosterone (T): 86.69 ng/dL (normal range, 75-400), dihydrotestosterone (DHT): 7.09 ng/dL (normal range, DHT decreases rapidly in the first week, then increases to 12-85 ng/dL between 30-60 days. Levels then decrease gradually to prepubertal values by seven months. Prepubertal children <3 ng/dL). When he was four months old, basal 17-hydroxyprogesterone (17-OHP) concentration was 1.4 ng/mL (normal range, 2 ng/mL), on ACTH stimulation test a peak cortisol response of 22.3 µg/dL and peak 17-OHP concentration of 4.9 ng/mL were observed and adrenal insufficiency was excluded. His hormonal evaluation when he was eight months old showed: Serum T concentration of 12.9 ng/dL (normal range, <3-10); estradiol concentration of <1 ng/dL (normal range, <1.5); follicle‑stimulating hormone (FSH) concentration of 0.28 mIU/mL (normal range, 0.16-4.1), luteinizing hormone (LH) concentration of 0.03 mIU/mL (normal range, 0.02-7.0) and prolactin concentration of 10.16 ng/mL (normal range, 3-18). After human chorionic gonadotropin (hCG) stimulation performed to assess testicular functions at the age of one year, serum T was 129.7 ng/dL (normal value, 65-250), and it was considered a sufficient response. Afterward, the patient discontinued his follow-up in the external center.

**Figure 1:**
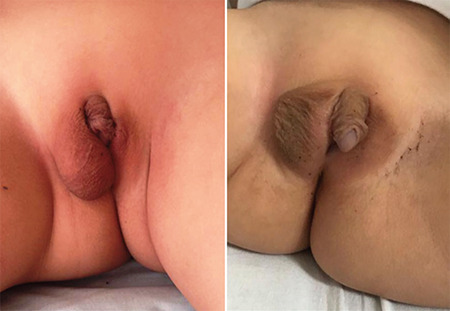
The genital photograph of the patient was taken at the age of three years and four months

At the time of writing the patient is three years and four months old. On physical examination body weight was 12.6 kg (-1.69 SDS), height was 89.5 cm (-2.4 SDS) and his head circumference was 50 cm (-0.35 SDS). There was no dysmorphism, scoliosis or skeletal dysplasia. Asymmetry was observed in his genital examination and phallus was 3.9 cm, cordis was observed, there was a single narrow urethral orifice opening to the phallus base. A 1 mL gonad was palpated in the labioscrotal fold on the right, a 1 mL gonad was palpated in the inguinal canal proximal on the left. In Figure 1, the external genital structure is shown. The most recent hormonal assessment of the patient revealed; serum T <1 ng/dL (normal range, <3-10); estradiol <1.2 ng/dL (normal range, <1.5 ng/dL); FSH 0.87 mIU/mL (normal range, 0.26-3.0), LH 0.53 mIU/mL (normal range, 0.02-0.3) and serum anti-Müllerian hormone (AMH) concentration of 19 ng/mL (normal range, 48.0-83.2). The hCG test was repeated with 1500 units of hCG given over three consecutive days. After the test, the serum T was 40 ng/dL (normal value, 65-250 ng/dL).

**Figure 2:**
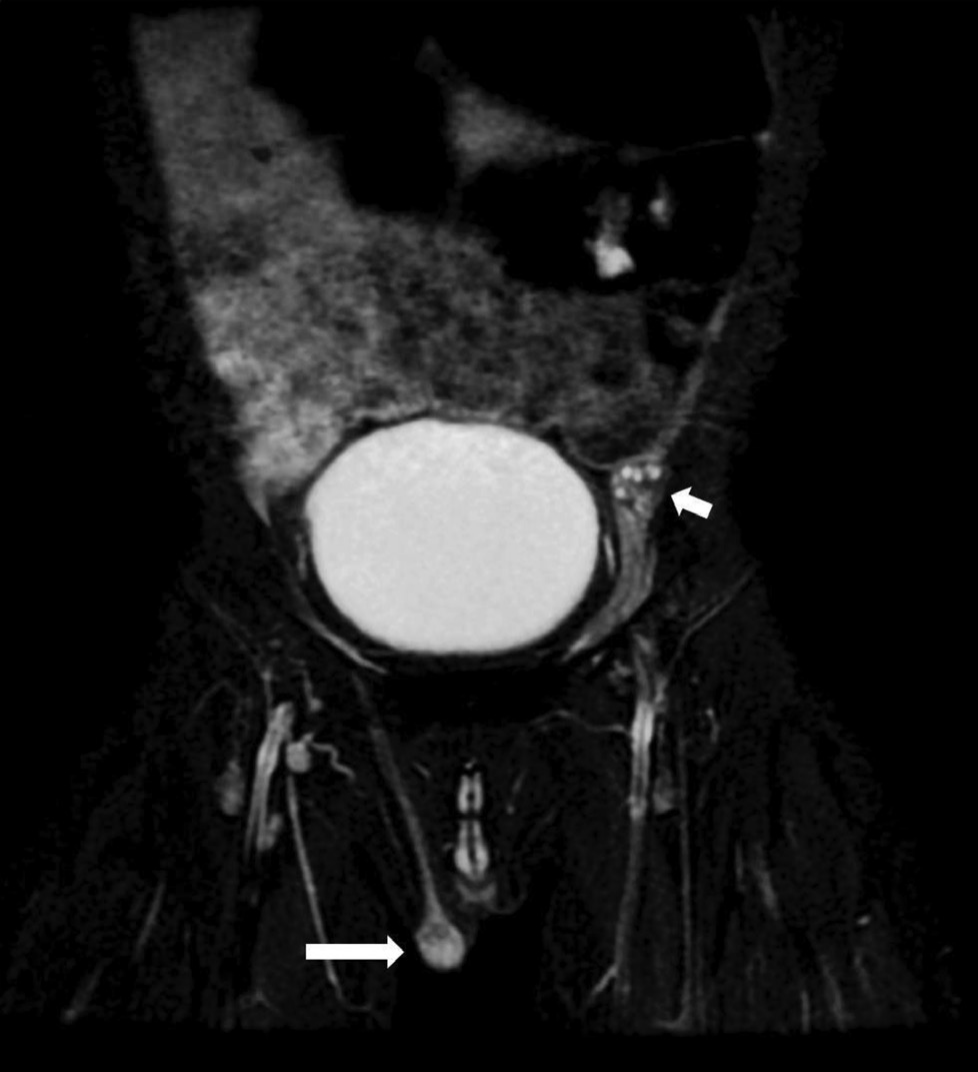
Coronal fat-suppressed T2-weighted image of the magnetic resonance sequence shows testicular tissue in the right scrotum. There is no testicular tissue in the scrotal area on the left. Ovarian tissue with millimetric follicular cysts is present in the left inguinal region

**Figure 3:**
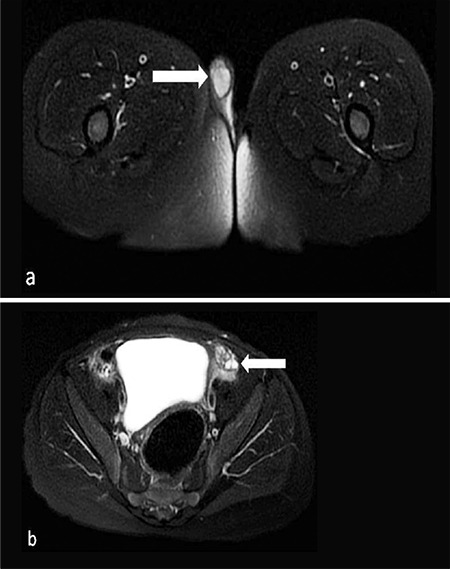
Axial fat-suppressed T2-weighted image of the magnetic resonance sequence shows testicular tissue in the right scrotum. Normal epididymis and spermatic cord are observed in association with this testicular structure (a). Ovarian tissue with millimetric follicular cysts is present in the left inguinal region (b)

**Figure 4:**
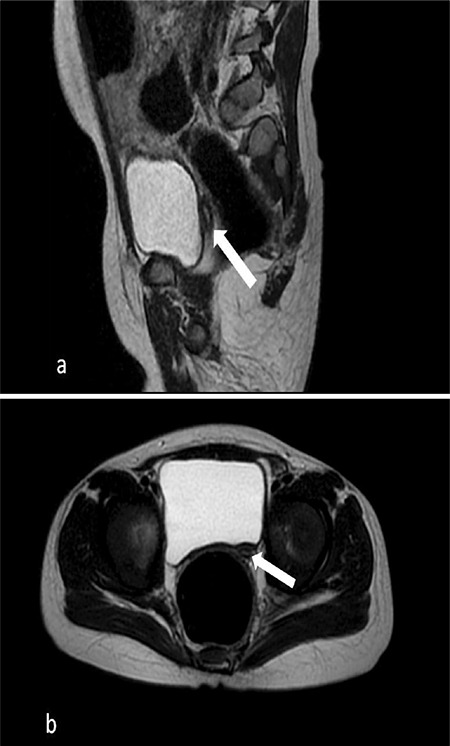
Rudimentaty uterus is shown on the sagittal T2 (a) and axial (b) T2-weighted magnetic resonance images

**Figure 5:**
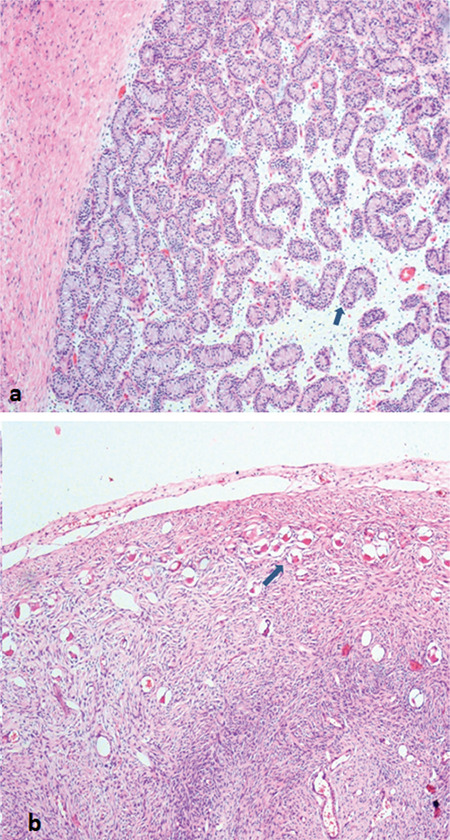
Testicular tissue with seminiferous tubules structure (a). Ovarian tissue containing follicle structures at different stages of maturation (b)

**Figure 6:**
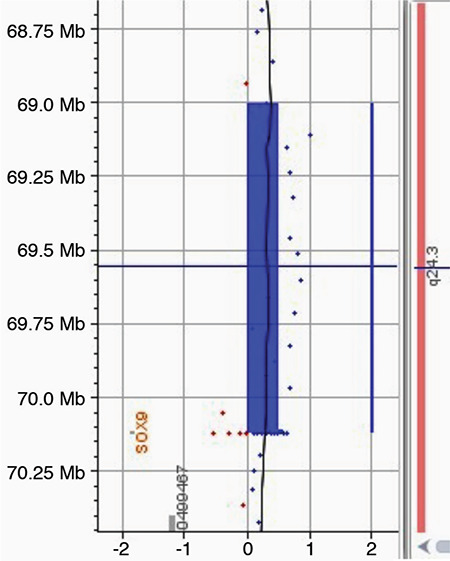
Patient result; in the comparative genomic hybridization study conducted on the peripheral blood DNA, a duplication of 1114 kb (Hg19 coordinates: chr17:69006280-70120619) was detected in the region of 17q24.3. This duplication covers the region from 1.1 Mb upstream of the *SOX9* gene (including the promoter region) to the 3’ UTR region

On pelvic ultrasound tissue was evident in the left inguinal canal with the appearance of ovarian tissue; dimensions were approximately 14x10x5 mm containing millimetric cystic areas. In addition there was testicular tissue with a homogeneous internal structure at the level of the hemiscrotum-labium majus on the right with dimensions 5x7x10 mm. In the left rectovesical area there was an apparent rudimentary structure with a front-back diameter of 5 mm. On abdominal and pelvic magnetic resonance imaging examination, the right testis located in the scrotum and its dimensions were approximately 13x8x11 mm. At the level of the inguinal canal proximal on the left, a structure was observed which again appeared to be ovarian tissue with the dimensions of 15x10 mm containing T2 hyperintense cystic areas that resembled follicular cysts (Figure 2, 3). There is a structure with the appearance of a rudimentary uterus, dimensions 7x5x17 mm, in the left posterior-inferior region (see Figure 4). A biopsy was taken from the wedge and central sections of the bilateral gonads. These gonadal tissues were examined histochemically. Histologically the right gonad was reported to be testis parenchyma containing a seminiferous tubule structure and the left gonad was reported to be ovarian tissue containing follicular structures at different maturation stages (see Figure 5). The *SRY* gene was not present in peripheral blood leucocytes by fluorescence *in situ* hybridization and polymerase chain reaction (PCR). Moreover, the *SRY* gene was found to be absent on analysis of the gonad biopsy material. In the array comparative genomic hybridization (CGH) study using DNA extracted from the peripheral blood sample (Agilent 8x60K array, Santa Clara, Ca, USA), a duplication of 1114 kb (Hg19 coordinates: chr17:69006280-70120619) was detected in the region of 17q24.3 (Figure 6). This duplication covers the region from the 1.1 Mb upstream of the *SOX9* gene (including the promoter region) to the 3’ UTR region. Furthermore, the array CHG confirmed that there were two copies of the X chromosome and the Y chromosome was absent. Unfortunately blood samples taken from the parents were insufficient and it could not be confirmed whether this variant was *de novo *as blood samples could not be taken again.

The patient was evaluated in the sex research commission. As a result of the psychiatric evaluation of the patient, it was reported that the patient’s selection of games and toys complied with the male sex and he selected playmates and clothes in compliance with the male sex according to the information given by the family, and as per the clinical conclusion reached, the patient’s development of sexual identity was male-oriented. As a result of the patient’s upbringing as a boy, and the family’s agreement with male-oriented corrective operations together with the medical and psychiatric evaluations, a decision was made to proceed with male-oriented corrective operations.

## Discussion

Ovotesticular DSD is defined as the presence of ovarian tissue with follicles and testicular tissue with seminiferous tubules in the same individual. Ovotesticular DSD is a rare DSD, with variable prevalence and karyotypes in different parts of the world. Although an ovotestis is the most commonly identified gonad in ovotesticular DSD, there may be an ovary on one side and a testis on the other (1). All the studies agree that 46,XX is the most common karyotype observed in blood samples of patients with ovotesticular DSD and the frequency varies between 65% and 90%. In the remaining cases, there is a Y chromosome (46,XY, 46,XX / 46,XY or other mosaic) that explains the development of the testicular tissue (3).

When the testicular tissue differs in a 46,XX *SRY*-negative gonad, two different mechanisms have been proposed: increased expression of the pro-testis genes or insufficient expression of the provarian/anti-testis genes (3).

The binary switch responsible for testicular development is the *SRY *gene, located on the short arm of the Y chromosome. The *SRY* protein contains a high-mobility group (HMG) domain and is encoded by a single exon gene. The *SRY* protein is expressed in pre-Sertoli cells, where it triggers a molecular switch to induce Sertoli cell differentiation, thus initiating the process of male sexual differentiation. A threshold *SRY* level must be achieved at a critical time during gestation to establish male sexual differentiation. Otherwise, the ovarian differentiation pathway is activated.

Available data suggest that steroidogenic factor-1 (*NR5A1*) promotes *SRY *expression (1).* SRY* expression is independent of the presence of germ cells. *SRY* increases the expression of the SRY-related HMG box-containing-9 (*SOX9*) gene. *SOX9* is a member of the SRY-related HMG domain gene family located at chromosome 17q24.3-17q25 (1). *SOX9* is expressed in various tissues including chondrocytes and testes. Furthermore, it is found in the bile duct, central nervous system, hair follicles, heart, lungs, pancreas, and retina (3). *SOX9* is highly expressed in Sertoli cells where it functions to promote Sertoli cell differentiation. Phenotype-genotype studies of humans and mice demonstrate that *SOX9* expression is a crucial step, downstream of *SRY*, in testis development. Upstream from the *SOX9* transcription start site, there appears to be a testis-specific enhancer element (*hTES*). *SOX9* then re-regulates fibroblast growth factor 9 and prostaglandin D2, and a positive feedback cycle is established to regulate *SOX9*, which gradually becomes independent of *SRY*. *SOX9* is responsible for the Sertoli cell specification, which in turn leads to initiation of testicular differentiation and AMH production is triggered (1,3).

Most of the *SOX9* duplications were identified in 46,XX testicular DSD patients (4). Firstly, Huang et al (8) (1999) reported an individual who had *SRY*-negative 46,XX testicular DSD and duplication of the 17q chromosome. To date, there have been only three studies of individuals with *SRY*-negative 46,XX ovotesticular DSD and duplication upstream of *SOX9*. Firstly, Benko et al (5) (2011) identified upstream duplications of *SOX9* in three cases with *SRY*-negative 46,XX ovotesticular DSD. These patients exhibited genital virilization to various degrees; one had gonads as bilateral ovotestis, one had ovarian remnant on the left and testis on the right, and the third patient had a streak gonad with partial ovarian differentiation on the right and ovotestis on the left. Molecular studies showed a different level of duplication in each patient. The region about 500 kb upstream of *SOX9* and covering 78 kb is accepted as the sex determination region (RevSex region). These authors asserted that the upstream region duplication of *SOX9* observed in 46,XX DSD patients had one or more regulating elements, which are critical for gonadal development (5). Secondly, Kim et al (7) (2015) reported two patients with 46,XX ovotesticular DSD. An upstream duplication of *SOX9* was found in both patients (including the RevSex region). Lastly, López-Hernández et al (4) (2018) conducted a molecular study on 10 unrelated patients with *SRY*-negative ovotesticular DSD. In only one patient, they found an heterozygosis duplication around 581 kb in the 5’ upstream region, including almost all the coding region of *SOX9*. This patient was six months old and brought up as a girl; one of the gonads was an ovary, the other one was an ovotestis.

*SOX9* duplication was also detected in 46,XX DSD studies. Vetro et al (9) (2015) analyzed 19 individuals with 46,XX DSD. In a patient with ambiguous external genitalia and 46,XX ovotesticular DSD, they detected a copy containing the gene-desert region upstream of *SOX9*, including the RevSex region. Recently, Ohnesorg et al (10) (2017) reported an individual with 46,XX with ovotesticular DSD with a heterozygous duplication upstream of *SOX9* encompassing a minimal region of 248 kb at 17q24.3.

Consequently, these studies demonstrate the importance of *SOX9* copies in male sexual differentiation with breasts and show that this is a key gene in testicular differentiation. We analyzed our *SRY *negative case with 46,XX ovotesticular DSD, the Y chromosomal sequence was not found in our patient. Therefore, testicular differentiation occurred in our patient in the absence of *SRY*. In the array CGH study a duplication of 1114 kb (Hg19 coordinates: chr17:69006280-70120619) was detected in the region of 17q24.3. This duplication covers the region from 1.1 Mb upstream of the *SOX9 *gene (including the promoter region) to the 3’ UTR region.

Duplication in the region of 17q that contains *SOX9* is not a common cause of testis development in subjects with *SRY*-negative 46,XX ovotesticular DSD. Seeherunvong et al (6) (2012) analyzed 30 *SRY*-negative people including 23 with 46,XX testicular DSD and seven with 46,XX ovotesticular DSD. They investigated the possible copies of *SOX9 *and duplication of the *SOX9* region in 17q was not detected in any subject. Rajender et al (11) used PCR and microsatellite analysis in order to examine a person with *SRY*-negative 46,XX testicular DSD. However, they could not detect a microduplication of *SOX9* (8). In a similar study conducted on twins, one of whom had 46,XX testicular DSD and the other one had 46,XX ovotesticular DSD, neither had *SOX9* duplication (12). Lastly, Temel et al (13) referred to a large family with nine members who had 46,XX testicular or 46,XX ovotesticular DSD. None of the affected individuals in the family group had *SOX9 *duplication.

Ovotesticular DSD requires the presence of seminiferous cords and ovarian follicles together with oocytes. The clinical picture does not differ from other types of DSD, there is a range extending from a male phenotype with mild hypospadias and cryptorchidism to a female phenotype with clitoromegaly and minor labial fusion. Internal genitals are usually associated with external virilization and Müllerian derivatives are observed in less virilized patients. Moreover, ultrasonographic evaluation may be deceptive as a diagnostic tool in neonates or infants. Histological analysis of the gonads is mandatory for diagnosis (3). The phenotype of our patient was male-dominant and he had a gonad as the testis and another gonad as ovarian tissue together with the rudimentary Müllerian structure.

T and AMH levels are generally between the normal male and female ranges (14,15). Estradiol levels can reflect the amount of functional ovarian tissues in neonates and teenagers. Gonadotropins may be increased or normal (12,14), the ovarian estrogen effect is reflected in these cases as the testicular tissue is dysgenetic in most conditions. Although the serum T concentration in mini-puberty and the serum T concentration following the hCG test, which was conducted at the age of one year, were adequate, the serum T concentration was substantially decreased in the later hCG test. In addition, the AMH concnetration was found to be under the age specific reference range. This suggested that function of the testicular tissue decreased as the patient became older.

Sex assignment is a problem in these patients and opinions are similar to those expressed in other forms of DSD (16). In patients with ovotesticular DSD, the ovarian tissue can be normal enough to produce oocytes. For this reason, a clinical aim may be the preservation of ovarian tissue and female assignment can be preferred. However, male sex was assigned to our patient. It was concluded that this selection resulted from the patient’s development of male sexual identity as a result of his social upbringing as a boy, the family’s willingness for male sex and psychiatric evaluation.

Partial gonadectomy requires specific interpretation. For children raised male, the ovarian part must be removed before the age of puberty, in order to prevent estrogen increase resulting in gynecomastia or other heterosexual pubertal development characteristics, and also the cystic folliclular complications that might emerge as a response to high FSH. In rare cases, it has been reported that male patients with a medical history of hypospadias and cryptorchidism presented with cyclic haematuria in puberty (17). In patients raised female, testicular tissue must be removed to prevent virilization in puberty. Regarding the risk of tumor growth in the testicular part, it was reported to be low, even if the tissue was dysgenetic, possibly because the Y chromosomal sequences are not available (18). For our case, a male-oriented corrective operation was planned due to the male sexual assignment.

## Conclusion

The *SOX9* gene is considered as the target of *SRY* and thus induces a gene expression resulting in the testicular assignment. Studies have demonstrated the importance of *SOX9* copies in male sexual differentiation with breasts and show that this is a key gene in testicular differentiation. Duplication in the region of 17q that contains *SOX9* is a rare cause of testis development in subjects with *SRY*-negative 46,XX ovotesticular DSD. Such DSDs are very rare and require a careful systematic and sensitive approach to diagnosis and management of the diagnostic and ethical challenges. This case is the first report from Turkey of a patient exhibiting *SOX9 *duplication in *SRY*-negative 46,XX ovotesticular DSD.
